# Long-term Efficacy and Safety of Sodium Oxybate in Treating Alcohol Use Disorder: A Systematic Review and Meta-Analysis

**DOI:** 10.2174/1570159X22666240902100058

**Published:** 2024-09-02

**Authors:** Letizia Biso, Andrea Spini, Francesco Petragnano, Roberto Maggio, Marco Scarselli, Marco Carli

**Affiliations:** 1 Department of Translational Research and New Technologies in Medicine and Surgery, University of Pisa, Pisa, Italy;; 2 Department of Medicine, Surgery and Neuroscience, University of Siena, Siena, Italy;; 3 Department of Biotechnological and Applied Clinical Sciences, University of L’Aquila, L’Aquila, Italy

**Keywords:** Sodium oxybate, alcohol use disorder, long-term treatment, efficacy, abstinence, safety, craving

## Abstract

**Background:**

Worldwide, three million deaths each year are reported due to the harmful use of alcohol. To date, only a few drugs have been approved for the treatment of Alcohol Use Disorder (AUD). This systematic review and meta-analysis aim to assess the long-term efficacy and safety of sodium oxybate (SMO) treatment in patients with AUD.

**Methods:**

We followed the PRISMA statement guidelines and searched PubMed and ISI Web of Science to retrieve the studies of interest. In total, 13 studies on long-term (>12 weeks) SMO administration in patients with AUD were included in this systematic review, and 7 were included in the meta-analysis.

**Results:**

Overall, the abstinence rate after 12 weeks of treatment was similar in the SMO and placebo groups, while it was significantly in favour of SMO compared to Naltrexone (NTX). The completion rate was similar in all three conditions. Mean corpuscular volume (MCV) levels favoured SMO over NTX, while Alcohol Craving Scale (ACS) scores did not favour SMO. The incidence of adverse reactions varied widely between studies.

**Conclusion:**

SMO in the chronic treatment of patients with AUD showed no superiority to placebo in our analysis of published RCTs, although many observational studies reported its beneficial effect in the long term. On the contrary, SMO was superior to NTX treatment on abstinence. The rate of study completion was similar in the three groups. Safety was not an issue in any of the studies included. Further studies are needed to better assess SMO efficacy and safety in the long term.

## 1 INTRODUCTION

Alcohol Use Disorder (AUD) is a common disorder associated with detrimental health consequences caused by alcohol consumption [1]. The prevalence of AUD is around 7-10% in Europe and in the US among the adult population [2, 3]. AUD and alcohol use in general are also common in adolescence, although at this age, binges are more frequent than daily alcohol consumption [4].

Worldwide, three million deaths each year are reported due to the harmful use of alcohol (5.3% of all deaths), and 5.1% of the world’s burden of diseases and injuries are attributable to alcohol, as measured in disability-adjusted life years [5]. Furthermore, there is a growing concern that the quarantine and social isolation associated with the COVID-19 pandemic have resulted in an increase in alcohol consumption and abuse [6].

Alcohol is responsible for a wide range of health problems, especially alcohol-related liver diseases that can cause an important proportion of the attributable morbidity and mortality. Analyses based on the general damage caused by different drugs (both to users and to other people) show that alcohol is the most dangerous one [7].

AUD is associated with changes in brain regions that are related to motivated behaviours and to the control of stress and emotions (*e.g*., the midbrain, the limbic system, the prefrontal cortex, and the amygdala), while reinforcement mechanisms (both positive and negative) contribute to the maintenance of drinking behaviour [8]. At the neurotransmitter level, dopamine, opioid peptides, serotonin, γ-aminobutyric acid (GABA), and endocannabinoids mediate the positive reinforcing effects of alcohol, while corticotropin-releasing factor, glutamatergic systems and down-regulation of GABA transmission mediate the negative reinforcement [9].

The treatment of AUD is complex and multidisciplinary, involving both pharmacological and behavioural approaches [10], with the goal of reaching total abstinence or at least having a significant reduction in alcohol consumption (harm reduction) [11].

Few drugs have been approved in Europe and in the US for the treatment of AUD. Currently, opioid antagonists naltrexone (NTX) and nalmefene are used as anti-craving drugs, along with the glutamate NMDA receptor antagonist acamprosate and the aversive agent disulfiram, an inhibitor of the enzyme acetaldehyde dehydrogenase [12-15]. Additionally, the sodium salt of γ-hydroxybutyric acid (GHB), sodium oxybate (SMO), has been approved for AUD treatment in Italy and Austria, specifically for the alcohol withdrawal syndrome (AWS) [16]. The Italian Medicines Agency (AIFA) has approved SMO for AWS only for the first 7-10 days of treatment, while the Austrian Federal Office for Safety in Health Care (BASG) is also considered a possible treatment extension to longer periods. In other European countries, Canada, and the US, its use is limited to narcolepsy with cataplexy, and SMO use is highly regulated in the US, being a schedule III-controlled substance [17].

Alcohol acts upon different neurotransmitter systems, but one of its main effects is GABA, the major inhibitory neurotransmitter in the central nervous system (CNS). GHB, whose sodium salt is SMO, is an endogenous short-chain fatty acid present in the CNS, and it is an analogue of GABA [18].

Few authors have focused on the efficacy and safety of SMO in maintaining abstinence from alcohol consumption in long-term trials [19-23]. SMO has been reported by some authors as an effective, well-tolerated, and safe treatment for both withdrawal and relapse prevention treatment in patients with AUD [24]. Some studies have shown its efficacy in reducing alcohol craving [25, 26].

Despite some evidence of efficacy, SMO use in clinical practice is limited since not all authors agree with these findings, and they have expressed concerns about its safety and efficacy. GHB has also gained an infamous reputation as one of the “date rape drugs” because of its ability to induce anterograde amnesia [27]. Moreover, it has emerged as a popular and potentially addictive party drug with a high risk of abuse. As a matter of fact, this substance could produce euphoric, relaxing, and sexually stimulating effects [28]. Especially when used improperly, GHB can potentially cause several adverse effects, including euphoria, ataxia, nystagmus, nausea, somnolence, aggression, hallucinations, seizures, loss of consciousness, and CNS depression that can lead to coma [29]. It is crucial to assess these potential undesired effects of SMO intoxication when considering this treatment because overdose in severe cases can lead to death; however, many studies have shown that SMO overdose outcome is usually non-fatal, as long as proper medical care is provided [30].

Because SMO is not used worldwide, not many studies have assessed its efficacy and tolerability in AUD long-term treatment. With this systematic review and meta-analysis, we aim to update the long-term efficacy and safety of SMO, considering the recent randomised clinical trials (RCTs) on the topic.

The primary objective of this systematic review and meta-analysis was to evaluate the long-term efficacy of SMO in patients with AUD. The secondary objectives were to assess the possible effects of craving for alcohol and to ameliorate the most common haematological parameters related to AUD. We also assessed the presence of adverse reactions and the risks for potential SMO craving and abuse.

## 2 METHODS

### 2.1 Study Design

The systematic review and meta-analysis protocol was registered on the PROSPERO website (CRD42021242091). This study was conducted according to the Preferred Reporting Items for Systematic Reviews and Meta-Analyses (PRISMA) [31].

### 2.2 Search Strategies

We searched PubMed and ISI Web of Knowledge for retrieving the studies of interest. Articles that were published before 31 December 2023 were considered suitable for inclusion. The search strategy was composed of three sets of keywords respectively related to the concepts “*alcohol*”, “*sodium oxybate*”, and “*clinical study*” (the full strings are available in Table **S1**). The snowballing search was also conducted to retrieve additional papers of interest by examining the references cited in the included articles and in the excluded reviews that were retrieved from the search strategy.

### 2.3 Eligibility Criteria

Studies including patients using SMO to prevent relapses and control cravings in alcohol addiction were included in this systematic review. Randomised clinical trials, cohort, prospective, and retrospective studies were considered suitable for inclusion in the systematic review, while only randomised trials were included in the meta-analysis. In order to include only maintenance and not AWS treatment studies, we decided to exclude studies that lasted less than 12 weeks. We included all studies that used AUD terminology, but also DSM-IV-TR and older terminology (“alcohol dependence” and similar terms that are no longer in use in modern classifications).

### 2.4 Study Selection

Two authors (MC and LB) screened all titles and abstracts of the references retrieved. Potentially relevant studies were further assessed through examination of full texts. The reviewers worked independently, in parallel, and blinded each other. Disagreement between the two reviewers was solved through discussion with a third author (AS). As for published studies, eligible studies had to be written in English, and studies with no full-text available were excluded.

### 2.5 Data Extraction

The following information was extracted:

- Study characteristics: phase, blinding, inclusion/ exclusion criteria, number of patients included per arm.
- Patients’ characteristics: mean age by arm, number of females, median follow-up time (range).
- Exposure: type of drug, dose/frequency, combined regimens, treatment duration.

Outcome: number of abstinent, trial completers, mean Alcohol Craving Scale (ACS), mean corpuscular volume (MCV), mean gamma-glutamyl transferase (GGT), mean aspartate aminotransferase (AST), mean alanine aminotransferase (ALT), adverse drug reactions (ADRs), and heavy drinking per arm. The abstinence rate was considered as the proportion of abstinent patients at the end of follow-up and used as a clinical parameter for efficacy [24]. Information was collected in a specific data sheet by MC and was validated by a second researcher (LB). Disagreement between the two reviewers was solved through discussion with a third researcher (AS).

### 2.6 Quality Assessment

Version 2 of the Cochrane risk-of-bias tool for randomised trials (RoB2) [32] was used to evaluate the quality of included randomised trials. Five items were considered, namely randomisation process, deviations from intended interventions, missing outcome data, measurement of the outcome, and selection of reported results. The overall risk of bias was assigned based on the RoB2 guidelines [33]. For non-randomised studies, the ROBINS-I tool [34] was used to assess seven domains: confounding, selection of participants, classification of intervention, deviations from intended interventions, missing data, measurement of outcomes, and selection of the reported results. The risk of bias coding was initially assessed by one researcher (LB) and then confirmed by a second investigator (AS).

### 2.7 Statistical Analysis

Characteristics of the included studies were described by study type, drugs, age, and female per single arm. As for dichotomous outcomes, the effect was measured as an odd ratio (OR) with a 95% confidence interval (95% CI). Continuous outcomes were estimated through mean differences. The statistical heterogeneity of the included studies was determined using a Chi-squared test and quantified using the I^2^ statistic. The significance level was defined as 0.05. If heterogeneity was significant, *i.e*., *p* ≤ 0.05 or I^2^ ≥ 50%, then a random effects model was required. If *p* > 0.05 and I^2^ < 50%, then the included studies were not heterogeneous, and a fixed effects model was used. Meta-analyses were conducted using JASP (version 0.18.1).

## 3 RESULTS

### 3.1 Study Selection

**Fig. (1):**
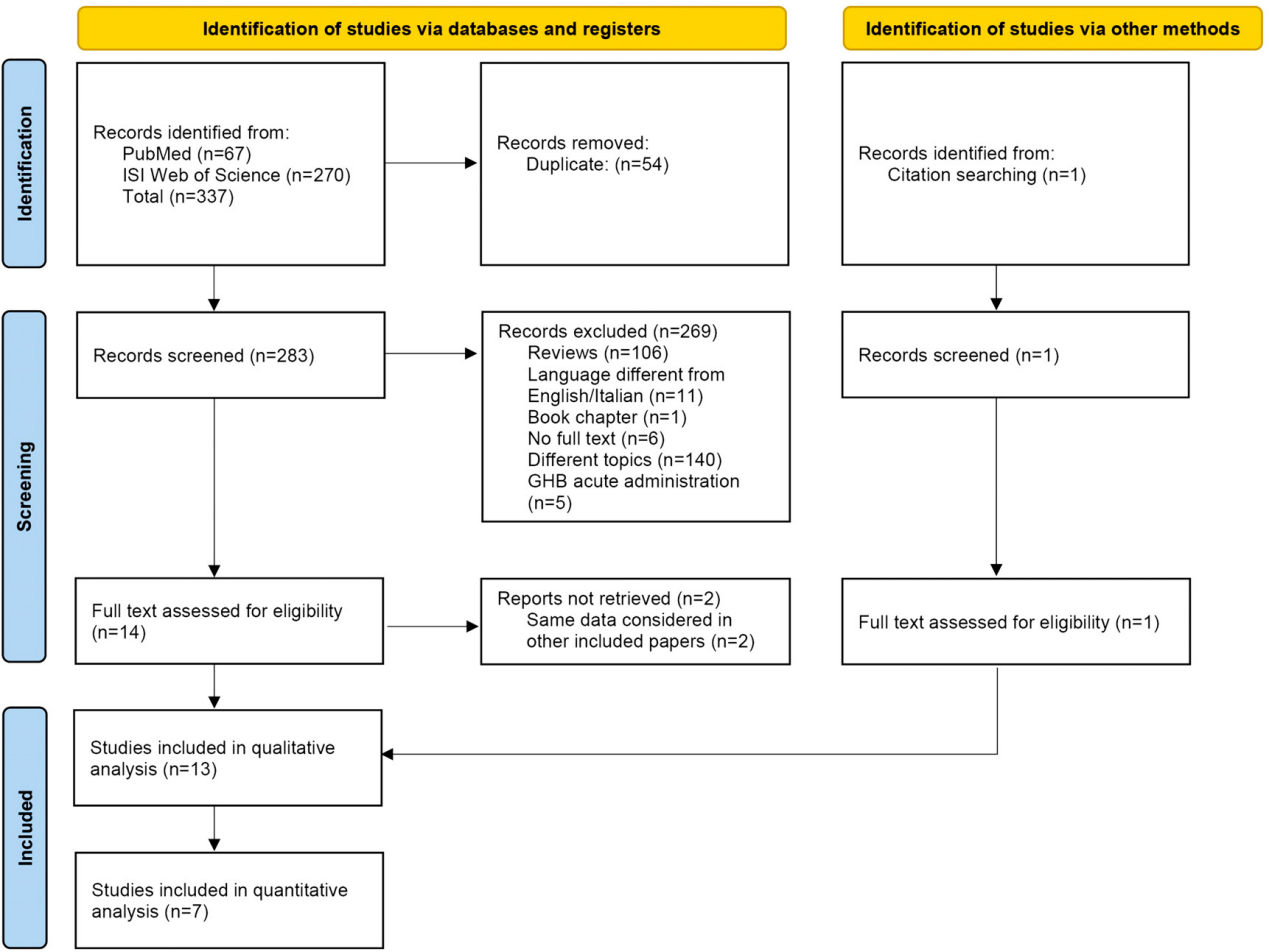
Flow diagram for screened records. From Page *et al*., 2021 (https://doi.org/10.1136/bmj.n71).

The PRISMA flowchart (Fig. **1**) shows the process of identification and selection of papers. In total, 67 and 270 records were identified from PubMed and Web of Science, respectively. One additional record was identified through other sources (citations in the reference list of a screened paper) and assessed for eligibility. After removing duplicate records, 283 articles were available for the screening of title and abstract. Fourteen studies were selected for full-text assessment. Two studies were excluded because they were short reports on the same sample of an included study. In total, 13 clinical studies on SMO chronic (>12 weeks) administration in patients with AUD were included in the systematic review. Among them, 5 studies were excluded from the meta-analysis. The RCT by Stella *et al*., 2008 [35] was not included in the meta-analysis because SMO is compared to escitalopram. The study by Caputo *et al*., 2011 [36] was not included in the meta-analysis because it is a prospective cohort study that evaluated SMO in the treatment of AUD in patients with and without psychiatric comorbidity. We excluded the study by Maremmani *et al*., 2011 [37] because it is a retrospective study without a control group, evaluating SMO response after 6 times in a day fractioning. Similarly, we also excluded the Maremmani *et al*. 2001 [22], Addolorato *et al*. 1998 [38], and Addolorato *et al*. 1996 [19] studies, being prospective cohort studies without a control group. In total, 7 RCTs were included in the meta-analysis.

### 3.2 Study Characteristics

Characteristics of the studies included in the systematic review are reported in Table **1** [19-22, 35-43]. Non randomised studies were clinical observations in patients with SMO administration for 12 weeks or longer [19, 22, 36-38]. One prospective cohort study analysed the efficacy of SMO on 179 patients treated for approximately 6 months on abstinence and ACS score [19] and reported a 78% abstinence rate and a reduction in craving. The same authors also evaluated the utility of SMO dose fractioning starting with 8 weeks SMO *ter in die* (tid) and, in non-responders, 8 more weeks with SMO six times per day [38]. The major fractioning of the drug caused a significant craving reduction, and 70.2% of non-responders achieved abstinence. The study by Maremmani *et al*., 2001 [22] evaluated the SMO efficacy in a 1-year prospective cohort study on 35 patients with treatment-resistant AUD. Twenty one patients completed the treatment and were considered responders, and between them 4 achieved abstinence and 5 reduced their alcohol intake. Another study [36] compared patients with and without psychiatric comorbidity treated for 12 weeks with SMO for their AUD. No difference was reported between the groups for abstinence and reduction of alcohol intake, while craving for alcohol was much higher in the group with psychiatric comorbidity. The study by Maremmani *et al*., 2011 [37] recruited patients who had previously failed a SMO treatment and treated them with SMO+disulfiram with a better retention in treatment. An open randomised trial was not included in the meta-analysis because it had escitalopram as a comparator [35].

As for the meta-analysis, seven studies were included [20, 21, 26, 39-42]: four studies were double-blind randomised [21, 26, 40, 41] while three were open randomised [20, 39, 42] for a total of 1085 subjects. The four double-blind RCTs evaluated different SMO doses *versus* placebo. The three open randomised studies evaluated SMO efficacy *versus* NTX [20, 42] and NTX or disulfiram [39]. In the latter study, the disulfiram group was excluded from the analysis. The duration of treatment was 12 weeks for five studies [20, 21, 39, 40, 42], 24 weeks for two studies [26, 41], and 52 weeks for one study [39]. The studies evaluated the efficacy of SMO in patients with alcohol dependence as defined by DSM-IV or DSM-IV-R criteria [20, 26, 39-42] or DSM-III-R criteria [21] since the terminology AUD has only been introduced with DSM-5. Patients included in all studies except one [21] had a period of untreated detoxification before randomisation of variable duration [from at least 3 days [40] to 30 days [26]. Common exclusion criteria were severe psychiatric disorders requiring medical treatment, a history of drug abuse or dependence, severe renal, hepatic, and respiratory problems, heart failure, epilepsy, and pregnancy. SMO was administered either with a fixed dose per day [40] or a per kilogram dose per day [20, 21, 26, 39, 42].

All the studies reported the number of abstinent patients and the number of trial completers at the end of follow-up. Craving symptoms were evaluated using the ACS at the beginning and at the end of treatment in four studies [20, 26, 39, 42]. However, for the study by Moncini *et al*., 2000 [26] the values are reported only in figures, and it was not possible to retrieve the exact data. Liver enzymes and MCV were reported in all the studies except one [26]. The study by Guiraud *et al*., 2021 [40] reports these haematological parameters only at study entry and not at the end of treatment.

### 3.3 Quality Assessment

Results of the quality assessment of the included studies in the meta-analysis are reported in Fig. (**2a**) and in the systematic review in Fig. (**2b**). The images were obtained using Robvis [43]. Overall, using the strict quality assessment tools RoB2 and ROBINS-I, none of the studies showed a low risk of bias. Two studies included in the meta-analysis [20, 42] scored an overall high risk of bias. We decided to include them anyway, as they represented most of the studies that assessed SMO efficacy against NTX, and they have been included in previous meta-analyses [24, 25]. The two recent studies by Guiraud and colleagues [40, 41] were the ones conducted with the most rigor; nonetheless, in both cases, the high number of missing data due to dropouts (37% and 58%, respectively) posed a problem in considering them at low risk of bias for the D3 domain.

### 3.4 Efficacy

#### 3.4.1 Abstinence: SMO versus Placebo

**Fig. (3):**
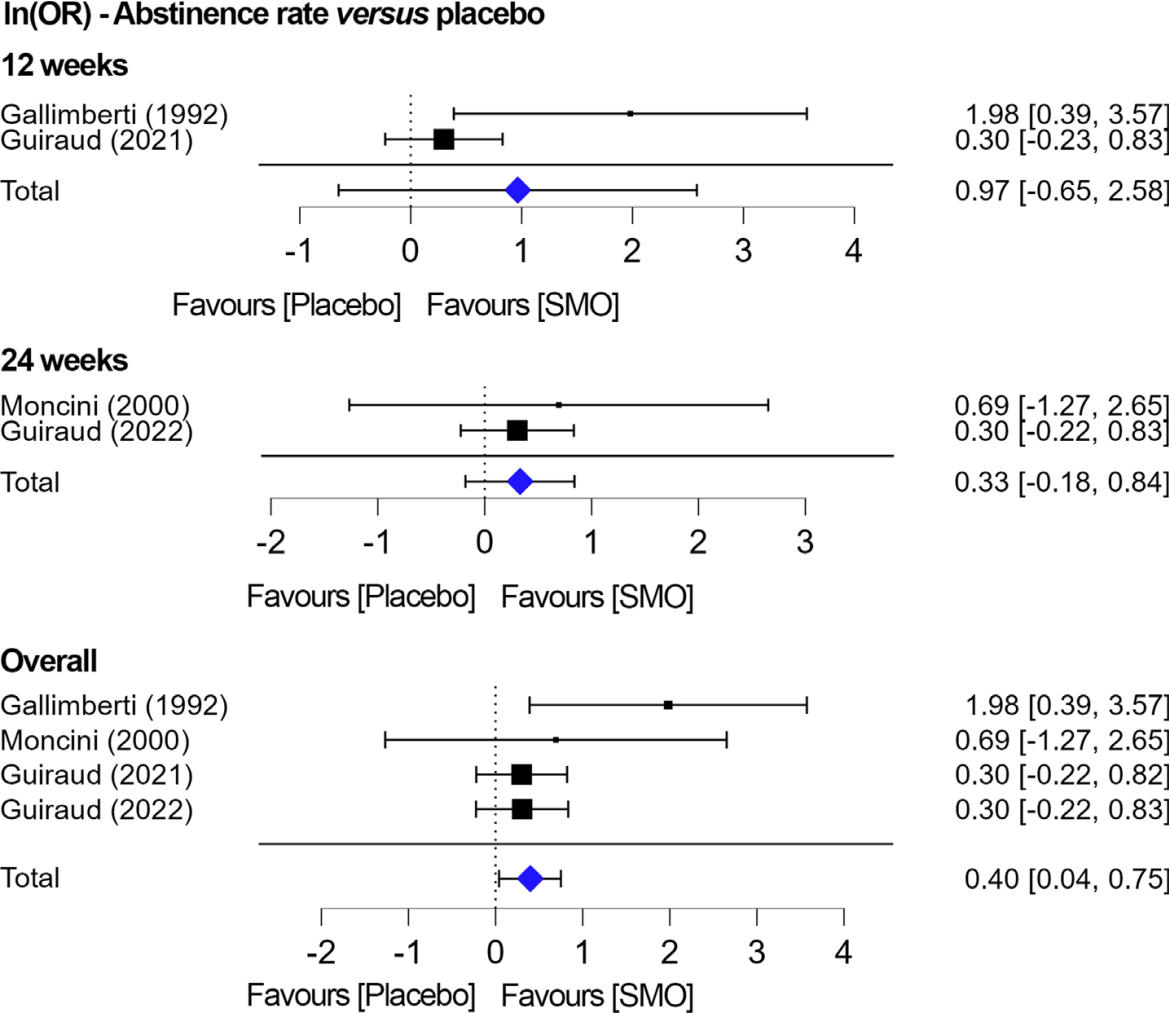
Efficacy of SMO *versus* placebo on abstinence at 12 and 24 weeks and overall.

Four studies compared the abstinence rates of SMO *versus* placebo [21, 26, 40, 41]. For the study by Guiraud *et al*. [41], the reported dose was slightly lower than the 50 mg/kg/day dose given in other studies (3.3 g/day for body weight up to 65 kg or 3.9 g/day for body weight higher than 65 kg) and we considered this study with dose uniformed limitation but valid for the treated *versus* untreated population. For the study by Guiraud *et al*., 2021 [40], SMO was administered at 0.75, 1.25, 1.75, and 2.25 g/day tid, and only data from the groups taking 1.25 and 1.75 g/day tid were pooled as comparable to doses administered in other studies. As for the pooled studies, two studies reported estimates at 12 weeks [21, 40], while the other two reported estimates at 24 weeks [26, 41]. At 12 weeks, a random model was used because of the high heterogeneity between the two studies, and no difference was reported between placebo and SMO (lnOR: 0.97; 95% CI: -0.65-2.58) (Z = 1.17, *p =* 0.24). At 24 weeks, a fixed model was used, and the lnOR was 0.33 (95% CI: -0.18-0.84) (Z = 1.27, *p =* 0.2). Overall, small heterogeneity was found among these latter two studies (I^2^ = 26%; *p =* 0.26), and the lnOD was 0.40 (95% CI: 0.04-0.75). When pooled together irrespective of study duration, SMO was significantly better than placebo (lnOR: 0.40; 95% CI: 0.04-0.75) in a fixed model (I^2^ = 0%; *p =* 0.25) (Fig. **3**).

#### 3.4.2 Abstinence: SMO versus NTX

**Fig. (4):**
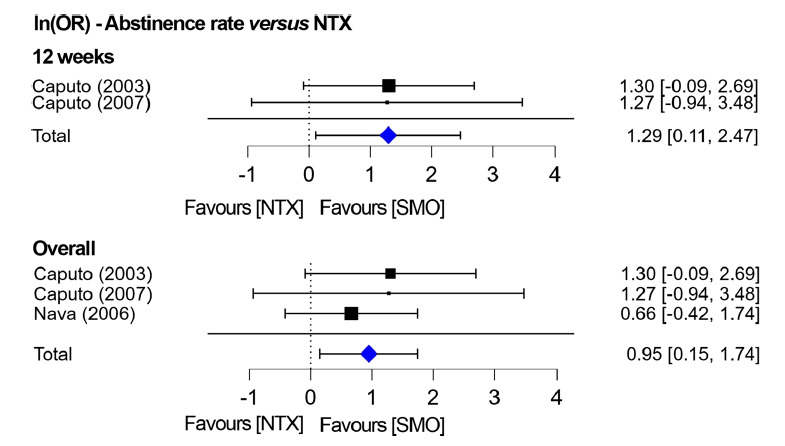
Efficacy of SMO *versus* placebo on abstinence at 12 and 24 weeks and overall.

Three studies compared the abstinence rates of SMO *versus* NTX [20, 39, 42]. Two studies reported estimates at 12 weeks [20, 42] while the other study at 52 weeks [39]. No high heterogeneity was found in the first stratification between the two 12-weeks studies (I^2^ = 0%; *p =* 0.98), and a fixed model was used. The results favoured SMO use over NTX use, with a *p =* 0.03, Z = 2.15 (lnOR: 1.29; 95% CI: 0.11-2.47). However, the study at 52 weeks reported no significant difference between SMO and NTX [39]. When pooled together irrespective of study duration, the abstinence rate was significantly in favour of SMO, with *p =* 0.02, Z = 2.34 (lnOR: 0.95; 95% CI: 0.15-1.74) (Fig. **4**).

#### 3.4.3 Completers: SMO versus placebo and SMO versus NTX

**Fig. (5):**
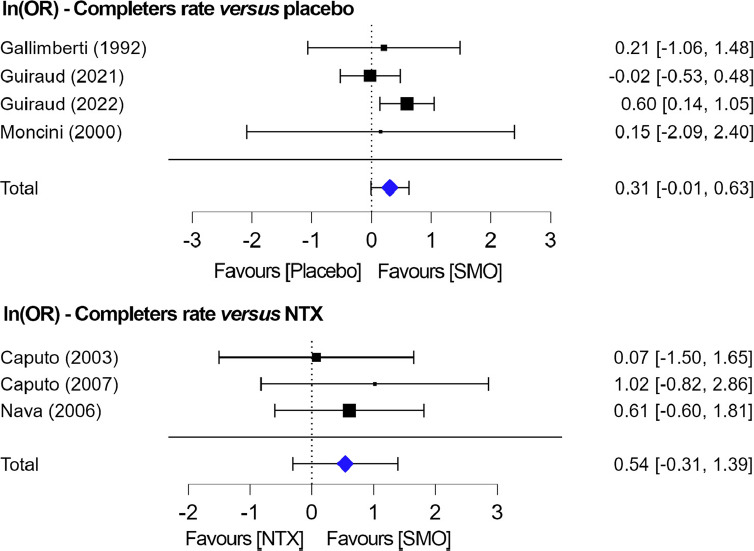
Efficacy of SMO *versus* placebo and *versus* NTX on study completers.

Four studies reported the number of participants who completed the treatment on SMO and placebo [21, 26, 40, 41]. All studies were pooled together with no difference in trial length (12 and 24 weeks). For the study by Guiraud *et al*., 2021 [40], only data from 1.25 and 1.75 mg/day tid were included. The heterogeneity was relatively low (I^2^ = 33, 2%, *p =* 0.36), and a fixed model could be used. Overall, the completion rate was similar in the two groups (*p =* 0.06, Z = 1.87; lnOR: 0.31; 95% CI: -0.01-0.63). Three studies evaluated SMO *versus* NTX [20, 39, 43], and no significant difference was reported between the two treatment groups (*p =* 0.21, Z = 1.24, lnOR: 0.54; 95% CI: -0.31-1.39) (Fig. **5**).

### 3.5 Other Outcomes

Other outcomes, such as ACS and MCV, were evaluated. Two studies reported ACS values comparing SMO *versus* NTX [39, 42]. High heterogeneity was found between these two studies, which reported results at different follow-up periods of 12 or 52 weeks (I^2^ = 96%, *p <* 0.001). Overall, the results indicate that with the given data, there was not a statistically significant difference between SMO and NTX in terms of ACS scores (*p =* 0.196, Z = -1.29, ES: -1.69, 95% CI: -4.25-0.87) (Fig. **S1a**). As for MCV, in this case, the same two studies comparing SMO *versus* NTX at 12 and 52 weeks reported better results for SMO [39, 42] (*p =* 0.02, Z = -2.32, ES: -1.23, 95% CI: -2.27-0.19) (Fig. **S1b**). The heterogeneity was high for this parameter (I^2^ = 79.98%, *p =* 0.025).

Obsessive Compulsive Drinking Scale (OCDS) scores were not meta-analysed because they were only used in one study [40]. In that study, the group receiving 1.75 g/day tid showed a significant end-of-treatment reduction in OCDS subscale for compulsive and resistance scores compared to placebo, while no significant difference was reported for other treatment groups. Finally, different studies reported heavy drinking or relapse into heavy drinking using different parameters, so we did not perform a meta-analysis on it. Relapse into heavy drinking is defined as “5 or more drinks on one occasion for men and 4 or more drinks on one occasion for women” [44]. This was reported by three studies [20, 39, 42], which described more frequent relapses in heavy drinking for SMO groups than NTX groups [20, 42], except for one study [39]. Another study reported a reduction in the number of heavy drinking days, with a significant difference for the 1.75 g/day tid (but not other groups) compared to placebo [40]. Lastly, the study by Gallimberti *et al*. [21] analysed the mean daily drinks, which decreased significantly in SMO-treated patients.

### 3.6 Adverse Drug Reactions

Not all the 13 studies included in the systematic review reported ADRs for the treated patients. Two studies [37, 38] did not assess ADRs at all, while a third [22] did not report them. In the study by Addolorato *et al*. [19], only percentages of the recurrence of ADRs are reported, but it is unclear if those percentages refer to the total number of recruited patients or to the completers. Table **2** shows the most common ADRs reported by patients during the treatment.

In general, the number of patients who showed ADRs during the studies greatly varied, ranging from more than 70% [40] to 3.5% [36].

The most common ADRs in the SMO groups were dizziness (5-27%), headache (1.5-23.5%), vertigo (3-27.7%), and fatigue (5.8-12.6%). Of all studies, only one [40] reported nasopharyngitis as an ADR despite being common (reported by 47 patients, between 7.8% and 15.8%, depending on the group).

Less common ADR included gastrointestinal disturbances, such as nausea (3-22%), diarrhoea (2.9-5.9%), vomiting (1.9%), somnolence (1.3-10.7%); insomnia (3.9-11.8%); and increased anxiety (3-6.8%).

The study by Guiraud *et al*. [41] also reported AWS (1 patient taking SMO and 2 patients taking placebo), delirium tremens (2 patients in the SMO group), and alcoholism (found in 3 patients in the placebo group), while those symptoms were not assessed as ADRs by other authors.

Importantly, patients receiving placebo also showed ADRs in percentages ranging from 7% [21] to 74.3% [40].

### 3.7 Long-term SMO Craving and Abuse

SMO craving and abuse were evaluated by some of the studies [19, 20, 26, 38, 40, 41]. Patients in both SMO and placebo groups reported comparable SMO craving levels on a 0-100 scale, with SMO = 38.21 ± 2.93 and placebo = 37.98 ± 3.40 [41]. The study by Guiraud *et al*., 2021 [40] reported no SMO abuse cases, and patients both in the SMO and in the placebo groups showed similar craving scores on a 0-10 scale (5.7 ± 5.1 in the SMO group; 5.7 ± 5.5 in the placebo group). In the study by Caputo *et al*. [20], two patients reported craving for SMO, but they both showed no withdrawal effects at the interruption of the treatment and maintained a reduction in alcohol consumption.

The study by Moncini *et al*. [26] did not directly report craving or abuse for SMO in their samples. However, they reported four (1.1%) total cases of SMO abuse out of 354 treated patients in their clinical centre between 1992 and 1995. Craving was not assessed in Addolorato *et al*. [38], but the study reported an absence of patients who abused SMO. Eleven patients out of nineteen, on the other hand, reported craving for SMO in the study by Addolorato *et al*. [19].

## 4 DISCUSSION

To the best of our knowledge, this is the most comprehensive and updated systematic review and meta-analysis evaluating the efficacy of long-term SMO treatment (>12 weeks) in patients with AUD. A precedent Cochrane Review meta-analysis on the topic dates back to 2010 [25], but it does not include the two most recent and numerous RCTs [40, 41]. In 2018, an attempt to pool data together from RCTs was published in a subsection of a review [24]. However, the methodology did not follow the PRISMA guidelines, and only abstinence was reported as an outcome, irrespective of different treatment duration. The method used a fixed model but had no report on the heterogeneity of the included studies. A recently published network meta-regression analysis by Guiraud and colleagues assessed the moderating effect of population severity and duration of treatment on the effects of SMO used for AUD treatment. Their data on abstinence favoured SMO but only in specific population subgroups, especially in patients with high-severity AUD, while the effect sizes of SMO in patients with mild-severity AUD were small [45].

SMO treatment is currently used in AWS in several European countries [46]; however, SMO also showed its efficacy in promoting abstinence and preventing relapses in patients with AUD treated continuously after the resolution of AWS symptoms. We examined a total of 13 clinical studies on SMO administered to patients with AUD for more than 12 weeks and, among them, meta-analysed 7 randomised studies comparing the efficacy of long-term SMO treatment *versus* placebo or NTX. In our analysis, the abstinence rate was similar in both SMO and placebo groups at 12 weeks (lnOR: 0.97; 95% CI: -0.65-2.58). At 24 weeks, the overall risk of abstinence was also not statistically significant (lnOR: 0.33, 95% CI: -0.18-0.84). In the precedent Cochrane Review [25], the abstinence rate was higher in the SMO group at 12 weeks but not statistically significant at 24 weeks. To have a significant superiority of SMO *versus* placebo, all studies must be analysed together irrespective of treatment duration, as done by Van den Brink *et al*. [24].

When compared to NTX, SMO showed an abstinence rate significantly higher at 12 weeks (lnOR: 1.29; 95% CI: 0.11-2.47). However, the only study at 52 weeks by Nava *et al*. [39] reported no significant difference between SMO and NTX. In the precedent Cochrane Review [25], data were similar to our analysis at 12 weeks and non-significant at 52 weeks. However, the two studies evaluating SMO *versus* NTX efficacy at 12 weeks [20, 42] were at high risk of bias, so these results need to be carefully evaluated. On the abstinence rate, the recent network meta-regression, including population severity and treatment duration as covariates, reported the SMO treatment effect significantly dependent on population severity (RR: 3.16 in the high severity group compared to the mild severity group) but not on treatment duration [45]. This could be considered consistent with our finding of a lack of efficacy of SMO either at 12 or 24 weeks *versus* placebo, with duration not influencing the abstinence rate. Following these considerations, further studies with long-term SMO treatment specifically designed for subgroups of patients with AUD, such as those with high severity AUD, are probably needed to demonstrate the hypothesized SMO superiority over placebo. In fact, in studies for the treatment of AUD, high-severity populations showed 1) a lower placebo response and a higher effect size of the tested drugs than the mild severity population; 2) a lower placebo response associated with longer treatment duration [47]. To sum up, these elements could influence the difference between SMO *versus* placebo groups and clarify the efficacy of SMO in chronic severe AUD treatment.

Additionally, patients from placebo groups had a relatively high response to abstinence. In particular, in most recent studies, the proportion of abstinent patients at 12 [40] and 24 weeks [41] was 29.3% and 20% for placebo and 35.9% and 25.3% for SMO treatment, respectively. This could be related to the selective inclusion of patients who had a period of detoxification before trial initiation. In fact, some recent findings highlight how placebo response was higher in AUD patients who accomplished more than 14 days of abstinence before randomisation [24, 47, 48]. The abstinence duration before randomisation also influences the effect of SMO treatment in patients with AUD [24, 40, 47]. Moreover, a few studies have theorised that high levels of neuroticism might be related to a greater placebo response, although not all authors agree [49, 50]. Neuroticism has been associated with patients with AUD, in particular with heavy drinkers [51, 52].

SMO efficacy in abstinence is also reported by other non-randomised long-term studies where SMO treatment determined abstinence in 67.8% of patients at 8 weeks [38] and 50% at 12 weeks [36].

No indirect comparison of NTX versus placebo for abstinence was conducted because it was beyond the aim of this meta-analysis, which included only studies evaluating SMO *versus* placebo and SMO *versus* NTX.

Study completers were evaluated as an outcome of clinical benefit. Dropout rates in patients with AUD are high and may be associated with suboptimal symptom control [53]. The rate of study completers was similar in SMO and placebo, although there was a trend in favour of SMO (lnOR: 0.31; 95% CI: -0.01-0.63), while no significant difference was found when patients were treated with either SMO or NTX (lnOR: 0.54; 95% CI: -0.31-1.39). Also, in non-randomised papers, dropout rates were high, and poor adherence represented a major limitation to the treatment of patients with SUD. For instance, patients who previously failed treatment with SMO were treated with SMO+disulfiram, and at 6 months, 18 out of 52 (35%) patients had dropped out of the treatment [37]. In the study by Maremmani *et al*. [22], 14 patients out of 35 (40%) left the program before trial completion at 12 months. In a dose fractionation study by Addolorato *et al*. [19], on 154 patients treated with SMO tid for 8 weeks, 115 completed the trial. Again, the placebo effect on this clinical outcome was high.

Concerning craving evaluation, we did not find statistically significant differences between SMO and NTX in ACS values (ES: -1.69; 95% CI: -4.25-0.87), but it is important to notice that the two studies were highly heterogeneous. OCDS values were not meta-analysed, because only one study used this scale. Indeed, the efficacy of SMO in reducing craving in the long term has been shown in two observational studies included in the systematic review [19, 38]. A reduction in alcohol craving could be caused by the SMO interactions with the GABAB receptor, as GABAB agonists like baclofen were shown to reduce alcohol craving [54].

Liver enzyme values and MCV were also evaluated, and SMO was in favour of NTX. These findings are in favour of the role of SMO in reducing alcohol intake. However, it should be noted that AST, ALT, GGT, and MCV increments are not specific for AUD health-related consequences. Notably, only two studies [40, 41] evaluated the carbohydrate-deficient transferrin (CDT). CDT is an accurate and specific biomarker of alcohol consumption, with greater specificity than other parameters (*i.e*., GGT and MCV) in patients with liver disorder [55-57]. It would, therefore, be important to include this parameter in further RCTs assessing SMO efficacy.

Considering long-term safety, ADRs were reported in 7% to more than 70% of patients treated with SMO. The wide range of ADRs reported by different studies included in the systematic review could depend on patients’ numerosity, pre-existing clinical conditions that could alter SMO metabolism and effects, the researcher’s meticulousness in assessing ADRs, given that none of the studies included safety evaluation as a primary outcome. Most reported ADRs were of mild severity and included dizziness, headache, vertigo, fatigue, and nasopharyngitis; these findings were also shown in a recent narrative review by Antonelli and colleagues [58]. A recent systematic review on SMO ADRs in patients with AUD reported a good tolerability profile for the drug, which was further confirmed by a post-marketing assessment on almost 300,000 patients from Italy and Austria, with the main ADRs being transitory dizziness and vertigo [16]. These results are in line with post-marketing studies on SMO safety in patients with narcolepsy that have reported similar percentages of ADRs (10-55%), with nausea, dizziness, vertigo, somnolence, and headache as the most frequent [59-61]. It is to be noted that the therapeutic dosage of SMO in narcolepsy is 6-9 g/day for adults, which is higher than the dosage used in AUD [62].

Interestingly, in the studies included in the systematic review, 3.5-73% of patients receiving placebo showed ADRs, with a high prevalence in the most recent studies [40, 41]. On this topic, a correlation was described between certain personality traits and placebo/nocebo effects [63]. In particular, the nocebo effect seems correlated with high pessimism, anxiety, suggestibility, and pain-catastrophizing personality traits; those traits are often found in patients with AUD [64-66]. All those traits could negatively impact expectations of treatment, leading to more perceived ADRs in the placebo groups.

Concerning craving for SMO and SMO abuse, the risk is limited for studies included in the systematic review. Some sparse cases are reported, such as the four patients out of 354 included in the trial by Moncini *et al*., 2000 [26]. Some studies suggest that patients with borderline personality disorder or poly-drug addiction (*i.e*., cocaine or heroin) could be more at risk of developing craving and consequent abuse of SMO [16, 24, 67]. Moreover, higher rates of SMO abuse were described for patients taking more than 20 g/day [68], much higher than the dose used in AUD. To diminish the risk of abuse, clinicians could consider excluding some subpopulations, such as polydrug users and patients with borderline personality disorder, but specific studies should assess this claim.

### 4.1 Strengths and Limitations

Strengths of our systematic review and meta-analysis are the inclusion of all the known studies that assessed SMO efficacy in the long-term in patients with AUD and the strict adherence to PRISMA statement guidelines. Limitations include the small number, small size, and heterogeneity of the studies, except for the two most recent RCTs [40, 41]. However, in these two studies, the dropout rates were high, which is the reason why, even in this case, we should not consider the two RCTs at low risk of bias, as suggested by the RoB2 guidelines [33]. Some of the studies were clearly funded by SMO producers, while others do not disclose any financial support or conflict of interest.

Moreover, the studies that met the criteria for being included in a meta-analysis compared SMO to either placebo or NTX. One study [39] had a comparison group to disulfiram, but being the only group with this drug, it could not be meta-analysed. Thus, it would be important to compare SMO with other drugs used to treat chronic AUD other than NTX.

Another limitation is that most of the patients included in the studies were males. Compared to men, women tend to manifest fewer risk factors to develop AUD and to perceive more stigma related to alcohol consumption [69]. This could lead to avoiding seeking help [70, 71], resulting in the underdiagnosis of women with AUD.

## CONCLUSION

Although many observational studies reported the efficacy of SMO in the long-term treatment of patients with AUD, this is not demonstrated by data from existing RCTs. In fact, SMO is not superior to placebo in terms of abstinence rate, while it is better than NTX. The completer rates are also similar in SMO, placebo, or NTX groups. However, given the high placebo response and the possibility of better responses in patient subgroups, further studies are needed to support or discard chronic SMO use, particularly in subjects with high-severity AUD. As shown by our analysis, the overall quality of the existing studies raises some concerns about the results obtained, questioning the reliability of the conclusions of our current findings and previous existing reviews. Additionally, the good safety profile and the limited risk for SMO craving, abuse, or dependence support the use of this drug for the treatment of patients with AUD, and this was also reported in other reviews such as Antonelli *et al*. [58]. Indeed, given the limited number of trials on SMO for AUD maintenance treatment, and the public health burden that AUD constitutes worldwide, there is a great need for more studies to clarify the efficacy of this drug in the chronic setting.

## AUTHORS' CONTRIBUTIONS

The authors confirm contribution to the paper as follows: LB conceptualization, investigation, data curation, writing original draft; AS methodology, formal analysis, writing original draft; FP investigation, data curation; RM writing original draft, supervision; MS conceptualization, writing original draft, supervision; MC conceptualization, investigation, data curation, writing original draft.

## Figures and Tables

**Fig. (2):**
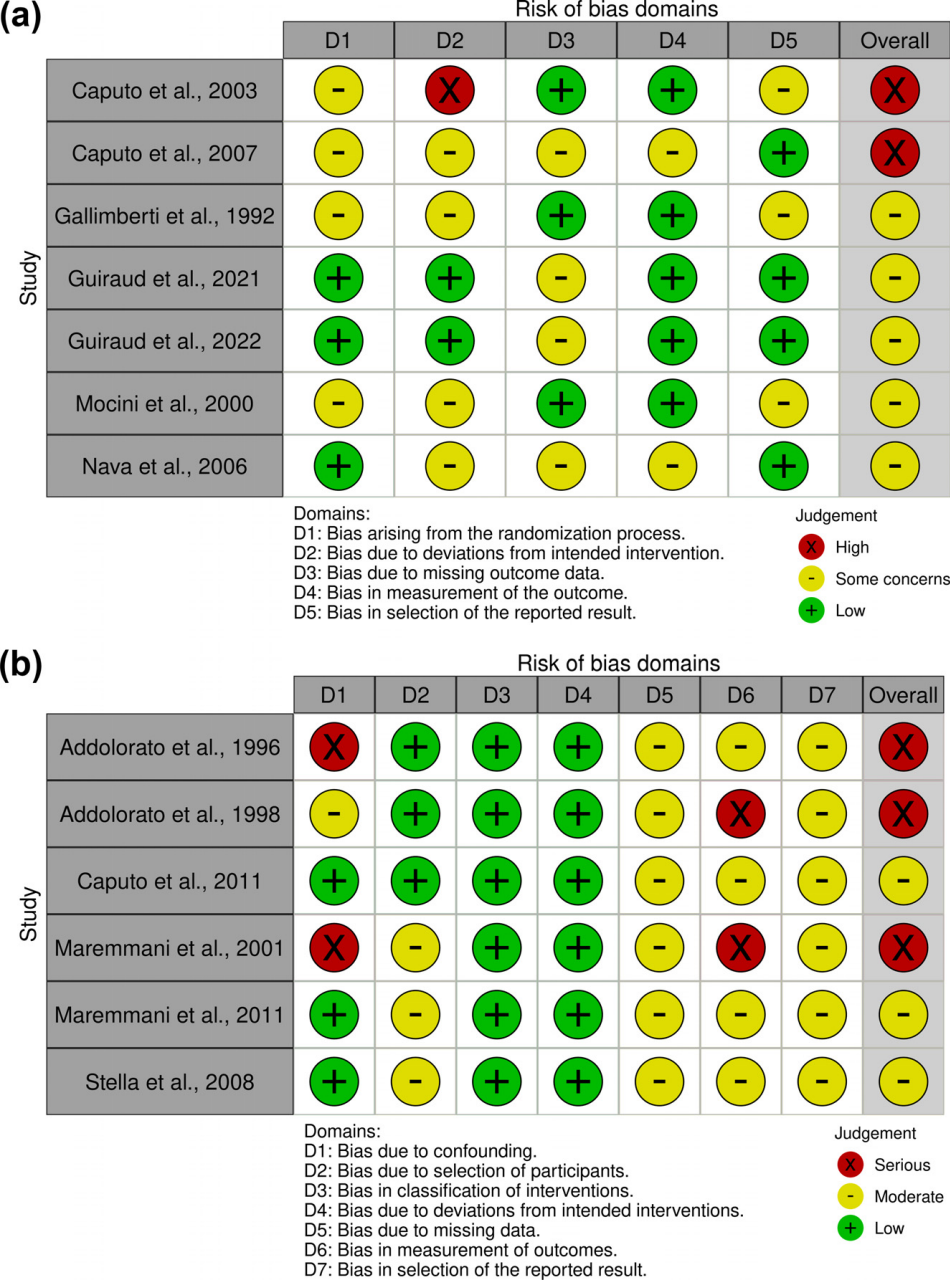
Quality assessment of the studies included in the meta-analysis (**a**) and in the systematic review (**b**).

**Table 1:** Characteristics of included studies in the systematic review assessing the efficacy of long-term SMO treatment.

**References**	**Study Type**	**Sample Size**	**Duration**	**Age (SD)**	**Gender**	**Groups**	**Outcomes**	**Included in the Meta-Analysis**
Guiraud *et al*., 2022 [41]	Double-blind Randomised	314	24 weeks	Group 1: 44.5 (9.8) Group 2: 44.3 (8.7)	Group 1: 129M; 31F Group 2: 121M; 33F	Group 1: placebo Group 2: SMO 3.3-3.9 g/day	n of abstinent; n of completers; MCV and GGT t0 and t1.	Yes
Guiraud *et al*., 2021 [40]	Double-blind randomised	511	12 weeks	Group 1: 48.3 (11.2) Group 2: 47.1 (11.9) Group 3: 47.4 (10.4) Group 4: 48.1 (11.6) Group 5: 47.7 (11.2)	Group 1: 67M; 32F Group 2: 77M; 22F Group 3: 75M; 24F Group 4: 77M; 22F Group 5: 76M; 24F	Group 1: placebo Group 2: SMO 0.75 g/day tid Group 3: SMO 1.25 g/day tid Group 4: SMO 1.75 g/day tid Group 5: SMO 2.25 g/day tid	n of abstinent; n of completers.	Yes
Maremmani *et al*., 2011 [37]	Retrospective	52	24 weeks	40 (11)	23M; 28F	Group 1: responders to SMO 50-100 mg/kg/day tid or six times per day Group 2: non-responders to SMO 50-100 mg/kg/day tid or six times per day	n of completers; retention in treatment; days of complete abstinence.	No
Caputo *et al*., 2011 [36]	Prospective cohort	48	12 weeks	Group 1: 47.3 (1.3) Group 2: 42.1 (11)	Group 1: 16M; 4F Group 2: 18M; 10F	Group 1: alcoholic patients with no comorbidity SNO 50 mg/kg/day tid Group 2: alcoholic patients with comorbidity SMO 50 mg/kg/day tid	n of abstinent; n of completers; relapse into heavy drinking; ACS t0, t1, t2, t3, t4; MCV, GGT, AST and ALT t0, t1, t2, t3, t4.	No
Stella *et al*., 2008 [35]	Open randomised	47	24 weeks	Group 1: 41 (13) Group 2: 39 (12) Group 3: 44 (13) Group 4: 43 (11)	Group 1: 8M; 3F Group 2: 8M; 4F Group 3: 8M; 4F Group 4: 9M; 3F	Group 1: escitalopram 20 mg/day Group 2: NTX 50 mg/day, escitalopram 20 mg/day Group 3: SMO 75 mg/kg/day five times a day, escitalopram 20 mg Group 4: NTX 50 mg/day, SMO 75 mg/kg/day five times, escitalopram 20 mg	n of abstinent; n of completers; relapse into heavy drinking; MCV, GGT, AST and ALT t0 and t1.	No
Caputo *et al*., 2007 [20]	Open randomised	55	12 weeks	Group 1: 47.8 (11.2) Group 2: 48.1 (10.8) Group 3: 48.1 (9.8)	Group 1: 16M; 4F Group 2: 14M; 4F Group 3: 13M; 4F	Group 1: SMO 50 mg/kg/day tid Group 2: SMO 50 mg/kg/day tid and NTX 50 mg/day Group 3: NTX 50 mg/day	n of abstinent; n of completers; relapse into heavy drinking; ACS t0 and t1; MCV, GGT, AST and ALT t0 and t1.	Yes
Nava *et al*., 2006 [39]	Open randomised	86	52 weeks	Group 1: 38.5 (7.9) Group 2: 40.8 (6.8) Group 3: 42.7 (4.7)	Group 1: 22M; 6F Group 2: 21M; 3F Group 3: 25M; 3F	Group 1: SMO 50 mg/kg/day tid Group 2: NTX 50 mg/day Group 3: disulfiram 200 mg/day	n of abstinent; n of completers; relapse into heavy drinking; ACS t0 and t1; MCV, GGT, AST and ALT t0 and t1.	Yes
Caputo *et al*., 2003 [42]	Open randomised	35	12 weeks	Group 1: 48.1 (8.5) Group 2: 49.5 (9.8)	Group 1: 14M; 4F Group 2: 13M; 4F	Group 1: SMO 50 mg/kg/day tid Group 2: NTX 50 mg/day	n of abstinent; n of completers; ACS t0 and t1; MCV, GGT, AST and ALT t0 and t1.	Yes
Maremmani *et al*., 2001 [22]	Prospective cohort	35	52 weeks	41.83 (11.55)	17M; 18F	Group 1: responders to SMO 25-100 mg/kg/day Group 2: non-responders to SMO 25-100 mg/kg/day	n of abstinent; n of completers.	No
Moncini *et al*., 2000 [26]	Double-blind randomised	17	24 weeks	46.4	13 M; 4F	Group 1: SMO 50 mg/kg/day Group 2: placebo	n of abstinent; n of completers.	Yes
Addolorato *et al*., 1998 [38]	Prospective cohort	154	16 weeks	48 (10.1)	97M; 57F	Group 1: SMO 50 mg/kg/day tid for 8 weeks + 8 weeks Group 2: SMO 50 mg/kg/day tid for 8 weeks + SMO 50 mg/kg/day six times per day for 8 weeks	n of completers; ACS t0, t1, t2; GGT t0, t1, t2.	No
Addolorato *et al*., 1996 [19]	Prospective cohort	179	24 weeks	46	131M; 48F	SMO 50 mg/kg/day tid	n of abstinent; ACS t0 and t1; MCV, GGT, AST and ALT t0 and t1.	No
Gallimberti *et al*., 1992 [21]	Double-blind randomised	82	12 weeks	Group 1: 38.1 (13.4) Group 2: 36.8 (15.6)	Group 1: 23 M; 13 F Group 2: 24 M; 11 F	Group 1: SMO 50 mg/kg/day Group 2: placebo	n of abstinent; n of completers.	Yes

**Abbreviations:** SMO: sodium oxybate; NTX: naltrexone; ACS: alcohol craving scale; MCV: mean corpuscular volume; GGT: gamma-glutamyl transferase; AST: aspartate aminotransferase; ALT: alanine aminotransferase; tid: *ter in die*; n: number.

**Table 2:** Recurrence of the most common adverse drug reactions (ADRs) across the studies.

**Study**	**Total Adverse Reactions**	**Headache**	**Dizziness**	**Vertigo**	**Fatigue**	**Naso-pharyngitis**	**Nausea**	**Vomiting**	**Diarrhoea**	**Anxiety**	**Somnolence/ Sedation**	**Insomnia**
Guiraud *et al*., 2022 [41]	Placebo: 32 (20%) SMO: 29 (18.8%)	Placebo: 3 (1.9%) SMO: 3 (1.9%)	Placebo: 8 (5.0%) SMO: 9 (5.8%)	NR	NR	NR	Placebo: 5 (3.1%) SMO: 4 (2.6%)	Placebo: 2 (1.3%) SMO: 3 (1.9%)	NR	NR	Placebo: 0 SMO: 2 (1.3%)	NR
Guiraud *et al*., 2021 [40]	Placebo: 75 (74.3%) SMO: 0.75g tid: 73 (71.6%) 1.25g tid: 73 (71.6%) 1.75g tid: 87 (86.1%) 2.25g tid: 81 (78.6%)	Placebo: 23 (22.8%) SMO: 0.75g tid: 24 (23.5%) 1.25g tid: 15 (14.7%) 1.75g tid: 18 (17.8%) 2.25g tid: 19 (18.4%)"	Placebo: 7 (6.9%) SMO: 0.75g tid: 7 (6.9%) 1.25g tid: 16 (15.7%) 1.75g tid: 25 (24.8%) 2.25g tid: 28 (27.2%)	Placebo: 3 (3.0%) SMO: 0.75g tid: 4 (3.9%) 1.25g tid: 9 (8.8%) 1.75g tid: 17 (16.8%) 2.25g tid: 12 (11.7%)	Placebo: 6 (5.9%) SMO: 0.75g tid: 6 (5.9%) 1.25g tid: 9 (8.8%) 1.75g tid: 11 (10.9%) 2.25g tid: 13 (12.6%)	Placebo: 13 (12.9%) SMO: 0.75g tid: 8 (7.8%) 1.25g tid: 13 (12.7%) 1.75g tid: 16 (15.8%) 2.25g tid: 10 (9.7%)	Placebo: 3 (3.0%) SMO: 0.75g tid: 4 (3.9%) 1.25g tid: 7 (6.9%) 1.75g tid: 8 (7.9%) 2.25g tid: 10 (9.7%)	NR	Placebo: 9 (8.9%) SMO: 0.75g tid: 6 (5.9%) 1.25g tid: 6 (5.9%) 1.75g tid: 5 (5.0%) 2.25g tid: 3 (2.9%)	Placebo: 7 (6.9%) SMO: 0.75g tid: 6 (5.9%) 1.25g tid: 5 (4.9%) 1.75g tid: 3 (3.0%) 2.25g tid: 7 (6.8%)	Placebo: 8 (7.9%) SMO: 0.75g tid: 8 (7.8%) 1.25g tid: 0 1.75g tid: 10 (9.9%) 2.25g tid: 11 (10.7%)	Placebo: 7 (6.9%) SMO: 0.75g tid:12 (11.8%) 1.25g tid: 6 (5.9%) 1.75g tid: 8 (7.9%) 2.25g tid: 4 (3.9%)"
Maremmani *et al*., 2011 [37]	NR	NR	NR	NR	NR	NR	NR	NR	NR	NR	NR	NR
Caputo *et al*., 2011 [36]	SMO w/o psychiatric comorbidity: 0 SMO with psychiatric comorbidity: 1 (3.5%)	NR	NR	SMO w/o psychiatric comorbidity: 0 SMO with psychiatric comorbidity: 1 (3.5%)	NR	NR	NR	NR	NR	NR	NR	NR
Stella *et al*., 2008 [35]	NR	NR	NR	NR	NR	NR	NR	NR	NR	NR	NR	NR
Caputo *et al*., 2007 [20]	SMO: 4 (20%) NTX: 2 (11.7%) SMO + NTX: 13 (72.2%)	SMO: 1 (1.5%) NTX: 0 SMO + NTX: 0	NR	SMO: 2 (3%) NTX: 0 SMO + NTX: 5 (27.7%)	NR	NR	SMO: 0 NTX: 2 (11.8%) SMO + NTX: 4 (22.2%)	NR	NR	NR	SMO: 0 NTX: 0 SMO + NTX: 1 (5.5%)	NR
Nava *et al*., 2006 [39]	SMO: 2 (7%) NTX: 2 (7%) DSF: 4 (12%)	NR	SMO: 0 NTX: 0 DSF: 4 (12%)	SMO: 2 (7%) NTX: 0 DSF: 0	NR	NR	SMO: 0 NTX: 2 (7%) DSF: 0	SMO: 0 NTX: 2 (7%) DSF: 0	NR	NR	SMO: 0 NTX: 0 DSF: 4 (12%)	NR
Caputo *et al*., 2003 [42]	SMO: 2 (11%) NTX: 6 (35%)	NR	SMO: 0 NTX: 1 (5.8%)	SMO: 1 (5.8%) NTX: 0	SMO: 1 (5.8%) NTX: 0	NR	SMO: 0 NTX: 3 (17.6%)	NR	NR	NR	NR	NR
Maremmani *et al*., 2001 [22]	NR	NR	NR	NR	NR	NR	NR	NR	NR	NR	NR	NR
Moncini *et al*., 2000 [26]	SMO: 2 (22.2%) Placebo: 2 (25%)	NR	SMO: 2 (22.2%) Placebo: 0	NR	NR	NR	SMO: 0 Placebo: 2 (25%)	SMO: 0 Placebo: 2 (25%)	NR	NR	NR	NR
Addolorato *et al*., 1998 [38]	NR	NR	NR	NR	NR	NR	NR	NR	NR	NR	NR	NR
Addolorato *et al*., 1996 [19]	NR	NR	NR	NR	NR	NR	NR	NR	NR	NR	NR	NR
Gallimberti *et al*., 1992 [21]	SMO: 9 (21.9%) Placebo: 3 (7%)	SMO: 2 (4.9%) Placebo: 1 (2.3%)	SMO: 4 (9.8%) Placebo: 1 (2.3%)	SMO: 3 (7.35%) Placebo: 0	NR	NR	SMO: 0 Placebo: 1 (2.3%)	NR	NR	NR	NR	NR

**Abbreviations:** SMO: sodium oxybate; NTX: naltrexone; tid: *ter in die*; NR: not reported.

## LIST OF ABBREVIATIONS

ACS: Alcohol Craving Scale
AST: Aspartate Aminotransferase
AUD: Alcohol Use Disorder
AWS: Alcohol Withdrawal Syndrome
CNS: Central Nervous System
GGT: Gamma-glutamyl Transferase
RCTs: Randomised Clinical Trials
SMO: Sodium Oxybate

## STANDARDS OF REPORTING

PRISMA guidelines and methodology were followed.

## SUPPLEMENTARY MATERIAL

PRISMA checklist and supplementary material are available as supplementary material on the publisher’s website along with the published article.
